# Maternal Exposure to Domestic Hair Cosmetics and Occupational Endocrine Disruptors Is Associated with a Higher Risk of Hypospadias in the Offspring

**DOI:** 10.3390/ijerph14010027

**Published:** 2016-12-29

**Authors:** Elodie Haraux, Karine Braun, Philippe Buisson, Erwan Stéphan-Blanchard, Camille Devauchelle, Jannick Ricard, Bernard Boudailliez, Pierre Tourneux, Richard Gouron, Karen Chardon

**Affiliations:** 1 Department of Paediatric Surgery, Amiens University Hospital, 80054 Amiens, France; buisson.philippe@chu-amiens.fr (P.B.); ricard.jannick@chu-amiens.fr (J.R.); gouron.richard@chu-amiens.fr (R.G.); 2 Department of Paediatrics, Amiens University Hospital, 80054 Amiens, France; braun.karine@chu-amiens.fr (K.B.); boudailliez.bernard@chu-amiens.fr (B.B.); 3 PériTox-INERIS Laboratory, Jules Verne University of Picardy, 80054 Amiens, France; erwan.stephan@u-picardie.fr (E.S.-B.); karen.chardon@u-picardie.fr (K.C.); 4 Department of Paediatrics, Creil Hospital, 60100 Creil, France; camille.devauchelle@ch-creil.fr; 5 Department of Paediatric Intensive Care Unit, Amiens University Hospital, 80054 Amiens, France; tourneux.pierre@chu-amiens.fr

**Keywords:** hypospadias, risk factor, endocrine-disrupting chemicals, cosmetics

## Abstract

Pregnant women are exposed to various chemical products at home and at work. Some of these products contain endocrine-disrupting chemicals (EDCs) such as cosmetics, pesticides, industrial chemicals, heavy metals, plastics or medications that could alter sexual differentiation and increase the risk of hypospadias. We evaluated maternal occupational and household exposures that could constitute risk factors for hypospadias. From 2011 to 2014, we enrolled 57 full-term newborns with hypospadias and three randomly selected controls per case (162 control newborns), matched for gestational age, from 11 maternity units in Picardy, France. Neonatal and parental data were collected at birth (personal characteristics, maternal lifestyle, and medical history). Maternal occupational exposure was assessed by a job-exposure matrix for EDCs from a job history questionnaire completed by mothers. Odds ratios (OR) and 95% confidence intervals (CI) were calculated with univariate and multivariable logistic regression, and adjusted for relevant covariates. Multivariate analysis showed a strong association between hypospadias and potential maternal occupational exposure to EDCs and maternal household use of hair cosmetics (OR 6.1, 95% CI: 1.1–34.9; OR: 9.6, 95% CI: 1.4–66.1, respectively). Our results suggest that maternal occupational exposure to EDCs is a risk factor for hypospadias and suggests a possible influence of household use of hair cosmetics during early pregnancy on the incidence of hypospadias in the offspring. A larger study with more accurate exposure assessment should evaluate the impact of EDCs in hair cosmetics on the incidence of hypospadias.

## 1. Introduction

Hypospadias is a congenital malformation in males due to insufficient virilisation of the genital tubercle that can impair male urinary and sexual functions. Insufficient development of ventral penile tissues contributes to varying degrees of severity of associated anomalies. The pathophysiology of hypospadias is complex and linked to endocrine, genetic, vascular, and environmental factors. However, only a few endocrinopathies are associated with hypospadias [1], and less than 25% of investigated cases of hypospadias present genetic mutations [2]. It has been hypothesised that hypospadias, cryptorchidism, and disorders of young adult males (low sperm counts, testicular germ cell cancer) may have a common origin in foetal life. This association of disorders, called testicular dysgenesis syndrome (TDS), could result from combinations of genetic, lifestyle, or environmental factors [3], including endocrine-disrupting chemicals (EDCs). The World Health Organization’s definition of an EDC is “an exogenous substance or mixture that alters function(s) of the endocrine system and consequently causes adverse health effects in an intact organism, or its progeny, or (sub)populations” [4]. EDCs mimic natural hormones, inhibit the action of hormones, or alter the normal regulatory function of the endocrine system [5]. Intrauterine exposure to EDCs could have antiandrogen effects with potential adverse effects on male development during early pregnancy, a period corresponding to the development of the external genitalia [6]. Over recent decades, many chemicals have been suspected to be endocrine disruptors (e.g., agricultural products, industrial chemicals, cosmetics, plastics, and medications) [5,7]. The large number of endocrine disruptors and their widespread presence in the environment result in ubiquitous exposure of the general population, including pregnant women. It has now been clearly documented that foetuses may be very sensitive to exposure to exogenous hormones [8]. The aim of this study was to evaluate the influence of occupational and household exposure of the foetus to EDCs on the incidence of hypospadias in a case–control study in the Picardy region. No measures have been implemented to limit the occupational or household exposure of women to endocrine disruptors, such as cosmetics, during the first trimester of pregnancy. Some authors have reported an association between prenatal exposure to EDCs present in chemical products or cosmetics and hypospadias, but without providing any details on the use of these products [2]. In order to propose prevention guidelines for pregnant women, we specifically studied targeted exposure to the household use of certain substances such as hair cosmetics, chemicals (e.g., glue), and pesticides (human and veterinary insecticides).

## 2. Experimental Section

### 2.1. Population

This study was approved by the Amiens University Hospital Ethics Committee (RCB No. 2010-A01491-38, Clinical Trial Registration number: NCT02805491). Data were derived from a case–control study conducted in Picardy (France) between March 2011 and March 2014 in 11 of the 16 maternity units that agreed to participate in the study. Informed consent was obtained from the parents. Cases were defined as newborns with hypospadias (Hypospadias Group). Three controls per case were selected randomly and matched for sex, gestational age, month of birth, and maternity unit of birth. Controls were male newborns without hypospadias (Control Group). Parents under the age of 18 years, parents deprived of their parental rights, and newborns with a life-threatening condition were excluded.

A total of 219 newborns (57 in the Hypospadias Group, 162 in the Control Group) were included. A first examination by a paediatrician was conducted at birth to establish the diagnosis. A hospital paediatric endocrinologist and paediatric urologist confirmed the diagnosis and severity at six weeks of life. The characteristics of newborns with hypospadias (severity of hypospadias, associated congenital anomalies) are presented in Table 1. Neonatal data were collected by means of a medical questionnaire. Low birth weight and length were defined as birth weight or length (adjusted for the mother’s age and the baby’s gender) below the fifth percentile relative to normative data for France [9].

Parental data were also collected on this questionnaire (parental age, height, weight and fertility disorders, maternal education level, medically assisted procreation, maternal medication during pregnancy, folate intake, family history of hypospadias, and undescended testis). Maternal endocrine medical history was also collected (age of menarche, oral contraception taken during the trimester preceding pregnancy, miscarriage, history of endocrine disease, long period of trying to become pregnant, hormone therapy). Maternal education level was defined as low (high school) and high (tertiary education).

### 2.2. Evaluation of Exposure

Foetal exposure was assessed on the basis of maternal exposure during the first trimester of pregnancy, the period corresponding to development of the external genitalia. Mothers admitted to the maternity unit were asked to complete a questionnaire before returning home. This questionnaire has been validated and used in a previous study [10] and was completed to investigate both pesticide exposure and exposure to cosmetics and chemical products.

Household, occupational, and environmental maternal exposures were then evaluated. Detailed data on lifestyle during the first trimester of pregnancy were collected: occupation; smoking; use of hair cosmetics (hairspray more or less once a week and colouring shampoo more or less once/first trimester) use and frequency of use of chemicals (paint, solvents, gasoline, ink, glue, and household products); and presence, number, and care of household pets (use of veterinary insecticides), use of human insecticides (ticks, lice, scabies), having a garden, living close to a field or a factory (<1 km).

Occupational exposures to EDCs were assessed using the job-exposure matrix (JEM) for EDCs developed by Van Tongeren [11]. This specific JEM for potential EDCs can be used to evaluate exposure to seven types of contaminants (pesticides, polychlorinated organic compounds, phthalates, alkylphenolic compounds, biphenolic compounds, heavy metals, and other substances) related to the mother’s occupation (348 possible jobs). Two independent coders used the JEM to review the job titles of mothers of cases and controls and assigned exposure to each type of EDC while blinded to the newborn’s case or control status. When the subject presented exposure to one of the seven categories, global occupational exposure to EDCs was considered to be positive. Any disagreement in the coding of the exposure between the coders was resolved by discussion.

### 2.3. Statistics

The population was characterised by descriptive analysis. The hypospadias group (57 newborns) and the control group (162 newborns) were compared. All statistical analyses were performed with SPSS Software (version 20.0, Chicago, IL, USA). Quantitative parameters were expressed as the mean and standard deviation (SD). Qualitative parameters were expressed as the number or percentage of the study population. The parents’ and infants’ characteristics and exposure to pollutants for cases and controls were compiled using univariate analysis (chi-square and Fisher’s exact test or unpaired *t*-test). The limit of significance threshold was *p* < 0.05. Associations between any exposure (i.e., “yes” to the question) and risk of hypospadias compared with no exposure were assessed between cases and controls by using the odds ratio (OR) and 95% confidence intervals (CI) calculated according to Woolf's method, with an alpha risk of 0.05. Multivariate linear regression and logistic regression analyses were conducted using forward selection, and covariates with a *p*-value < 0.15 in a univariate analysis were entered into the multivariate analyses.

## 3. Results

Table 2 and Table 3 summarize the characteristics of newborns and parents of cases and controls and the crude OR of their differences.

Term was a matching criterion for neonatal data and was therefore comparable in the two groups. Birth weight and length were significantly lower in cases than in controls. Consequently, low birth weight and length were also associated with a significantly increased risk of hypospadias (OR 7.4, 95% CI: 2.2–25.1); OR 3.8, 95% CI: 1.4–10.8), respectively). Moreover, mothers of cases were more likely than mothers of controls to be primiparous (47% vs. 31%, *p* = 0.03). 

No significant differences for maternal data (maternal age, body mass index (BMI), and educational level; Table 3) were observed between the two groups. The risk of hypospadias in the offspring was not significantly different among pregnant women who smoked (OR 0.9, 95% CI: 0.5–17.8), women who took an oral contraceptive pill (before and during the first trimester of pregnancy) or folate and/or vitamins, and women with a history of endocrine disease. This risk of hypospadias was significantly higher among pregnant women who took medications during pregnancy (OR 2.1, 95% CI: 0.9–4.6). Various medications were taken during pregnancy and sample sizes were too small to test for statistical associations between each medication and hypospadias. Mothers of cases used the following medications during pregnancy: levothyroxine (3 vs. 5 in the Control Group), insulin (2 vs. 0), β-blockers (1 vs. 2), antihypertensives (2 vs. 1), salbutamol (2 vs. 1), antiepileptics (1 vs. 0), antihistamines (1 vs. 0), steroids (2 vs. 0), aspirin (1 vs. 1), immunosuppressants (1 vs. 0), and methadone (1 vs. 0).

A family history of hypospadias appeared to be a risk factor (RF) for hypospadias in the offspring. 

Paternal weight and BMI (24.6 ± 3.40 vs. 26.3 ± 4.35, *p* = 0.039) were lower in the Hypospadias Group (Table 3).

Table 4 shows the characteristics of household, environmental, and occupational exposures. No association was observed between hypospadias and the use of any chemical products such as paint, solvents, gasoline, ink, glue, and household products (OR 0.6, 95% CI: 0.2–1.8). The use of hairspray and/or colouring shampoo (i.e., hair cosmetics) seemed to be linked to hypospadias (OR 1.8, 95% CI: 1.0–3.6). Mothers of newborns with hypospadias more frequently had pets and used veterinary insecticides during pregnancy (OR 2.2, 95% CI: 1.1–4.5 and OR 2.05, 95% CI: 1.0–4.1), respectively. Owning a garden was associated with an increased risk of hypospadias in the offspring (OR 2.3, 95% CI: 1.01–5.3). Among the 219 mothers of this cohort, 101 were housewives and 118 worked during their pregnancy. Global maternal occupational exposure to EDCs evaluated by JEM for EDCs was a significant risk for hypospadias (OR 3.1, 95% CI: 1.3–7.6).

Positive family history of hypospadias was excluded from the multiple logistic regression models because of the small sample size of certain groups (<5).

Two main risk factors for hypospadias were identified by multivariate analysis (Table 5).

After adjustment (neonatal birth weight, parity, paternal weight, and maternal folate and/or vitamin intake) household use of hair cosmetics (hairspray and/or colouring shampoo), and maternal occupational exposure to EDCs were the strongest contributors, among the covariates studied, to the presence of hypospadias in our study.

## 4. Discussion

This case–control study was designed to evaluate the influence of foetal exposure to occupational and household EDCs on the incidence of hypospadias in the Picardy region. We were specifically interested in certain household products in order to identify ways of preventing maternal exposure during early pregnancy. This study suggests that maternal occupational exposure to EDCs is a risk factor for hypospadias and the possible influence of home use of hair cosmetics during early pregnancy on the incidence of hypospadias in the offspring. 

The strengths of our study include confirmation of the diagnosis and its severity by two practitioners, an extensive interviewer-based questionnaire, and the use of a validated job-exposure matrix for EDCs. Most previous studies on hypospadias have been based on routinely collected registry data with limited information on potential risk factors and covariates and varying levels of quality control and completeness. Cases were included prospectively in this study.

However, our study has several limitations. The participation rate is unknown, as the number of women who declined to take part in the study was not recorded. Data about exposure were collected at birth. Consequently, quality of data concerning exposure during the first trimester could be altered by the retrospective nature of the question and by the fact that some mothers of newborns with hypospadias may have deliberately or unconsciously overestimated or underestimated exposures. The possible impact of this bias on exposure data cannot be determined. In case of overestimated exposures, this bias could have resulted in a false positive association and overestimation of the risk related to exposure that could constitute a confounder. We can also assume that mothers omitted to tick some boxes of the questionnaire simply because they did not use the product concerned by the question. As this assumption cannot be confirmed, an absence of response to this question was classified as missing data. Data were also considered to be missing when the answer was “do not know”, which explains the high rate of missing data for certain questions and which represents a significant bias in our study. Moreover, exposure to certain products could have been more specifically investigated. For example, exposure to household pesticides could have been more specifically investigated by asking the question “Do you use pesticides? How often?” The main weaknesses of our study remain the size of the cohort that prevented certain statistical analyses and the lack of information on paternal occupation. The results of this study therefore need to be interpreted cautiously.

In our region, 831 male infants were operated on for hypospadias between 2000 and 2008 [12]. The increasing hypospadias surgery rate in France suggests that the incidence of hypospadias is also increasing, but the real incidence and the growth of this incidence remain controversial [13,14]. Based on a review of the literature [15], the prevalence of hypospadias increased at the end of the 20th century and has subsequently remained stable. In any case, the prevalence of hypospadias remains considerable, affecting 1/250 live male newborns [16]. The severity of hypospadias is variable in the literature. In the past, 50% of cases were reported to be distal forms, 30% were middle, and 20% were proximal [17]. In our cohort, as in recent publications, distal forms accounted for about 80% of cases [2]. This increased rate of distal forms could be related to a better global knowledge of this malformation, leading to better screening by doctors and a greater demand for care by parents.

### 4.1. Neonatal Risk Factors

Low birth weight and length, and primiparity were significant RFs for hypospadias vs. controls. Prematurity was not studied, as only eight infants were born before 37 weeks of amenorrhoea. These factors, classically reported to be neonatal RFs for hypospadias [18], could be linked to placental dysfunction. Early placental dysfunction [19] could impact on foetal intake and hormonal regulation. The placenta is the main site of hormone production during virilisation of the genital organs. Decreased human chorionic gonadotrophin hormone (hCG) production could lead to impaired virilisation, resulting in hypospadias.

Antiandrogenic EDCs such as phthalates (mainly present in certain plastics, adhesives, and cosmetics) could also disrupt placental hCG differentially in males and females, with consequences for sexually dimorphic genital development. A recent study showed that first-trimester hCG levels of pregnant women may reflect the sexually dimorphic action of phthalates on placental function and genital development [20].

### 4.2. Parental Risk Factors and Medication

No association was observed between either maternal age or BMI and hypospadias. More advanced parental age was also not identified as an RF for hypospadias, as previously reported [16,21]. Discordant results have been published in the literature. Adams et al. [22] found no association between maternal obesity and hypospadias in a large prospective case-control study. These results were essentially adjusted to maternal age and parity. In contrast, Akre et al. [18] reported that hypospadias and high BMI before pregnancy could be related to placental dysfunction.

We found no impact of maternal endocrine factors (history of endocrine disease, hormone therapy, early age of menarche, long period trying to become pregnant). Oral contraceptives before or during early pregnancy were not associated with an increased risk of hypospadias, as previously published in a large case–control study [21]. No conclusion could be drawn concerning miscarriages, fertility disorders, and medically assisted procreation due to the small sample sizes. In the literature, the growing incidence of hypospadias has been frequently described as being associated with an increased incidence of fertility disorders [23].

Paternal weight and BMI were lower in the Hypospadias Group. Few data are available in the literature concerning the impact of paternal weight and no strong hypothesis can be proposed to explain this finding. According to Brouwers et al. [24], an increased risk of hypospadias was observed when fathers, but not mothers, used prescription drugs during the 3 months immediately prior to conception. On the contrary, we found a significant risk of hypospadias when mothers used prescription drugs during the first trimester of pregnancy. Several drugs identified as possible EDCs were taken by five mothers in the Hypospadias Group during pregnancy: valproic acid and corticosteroids with antiandrogenic properties and loratadine (antihistamine) with oestrogenic properties. The antiepileptic drug taken by one mother in the Hypospadias Group was a folic acid antagonist. Czeizel et al. [25] found that the use of folic acid antagonists in early pregnancy may increase the risk of urinary tract defects (not exclusively hypospadias), particularly among the infants of women who did not take a multivitamin supplement containing folic acid. It has been hypothesized that folate supplementation may prevent neural tube defects and, therefore, defects of midline structures such as the urethra [26]. Folic acid antagonists could exert an opposite effect [27]. Nevertheless, no statistically significant impact of maternal folate intake on the risk of hypospadias was observed in our cohort, as reported in previous studies [24]. An association was also observed between the risk of hypospadias and family history of hypospadias, supporting the hypothesis of a genetic aetiology together with the reported familial clustering and increased familial risk of recurrence [28]. Firstly, according to Kalfa et al. [28], the mode of inheritance is more likely multifactorial; secondly, susceptibility to environmental factors might depend not only on the EDCs themselves, but also on individual sensitivity, which is modulated by genetic background, including polymorphisms.

### 4.3. Domestic and Environmental EDCs Exposure

To ensure a more discriminant analysis, we specifically studied two hair cosmetics used at home during the first trimester of pregnancy (hairspray and colouring shampoo). This is the first study to demonstrate a link between maternal household exposure to these two hair cosmetics during early pregnancy and the incidence of hypospadias. A link with maternal occupational exposure to hair cosmetics has already been reported. Ormond et al. [29] reported a significant association between maternal occupational exposure to hairsprays and risk of hypospadias in a multivariate and adjusted analysis. Vrijheid et al. [30] reported a significantly increased risk of newborns with hypospadias among female hairdressers.

Hairspray and colouring shampoos contain substances whose dangerousness is regularly assessed. In 2006, the European Commission banned 22 hair dye substances to ensure the safety of hair dye products for consumers [31]. Phthalates and their metabolites present in hair cosmetics were incriminated. Many consumer products therefore contain specific members of this family of compounds, including cosmetics (diethyl phthalate) [32]. Consumer products containing phthalates can result in human exposure directly via contact and use, indirectly via leaching into other products, or general environmental contamination. Humans are exposed to such chemicals via ingestion, inhalation, and dermal exposure throughout their lifetime, including intrauterine life. Some phthalates are reproductive and developmental toxicants in animals and are suspected to be endocrine disruptors (EDs) in humans. Exposure assessment by modelling ambient data tends to suggest that exposure of the foetus and the child to phthalates is higher than in adults [32].

In fact, human in utero exposure to phthalates could contribute to TDS; the most important effect of phthalates is a decrease in foetal Leydig cell hormone production. As human phthalate exposure is ubiquitous during pregnancy [32,33,34], and as rat in utero phthalate exposure inhibits foetal testis hormone production, it can be hypothesized that genital anomalies dependent on foetal testis hormonal production could be caused by human in utero exposure to phthalates, as phthalates adversely affect the rat male reproductive system, inducing hypospadias, cryptorchidism, reduced testosterone production, and decreased sperm counts. Fisher et al. [35] studied the effects on male rats of in utero exposure to dibutyl phthalate (DBP) on gestational days (GD) 13–21. DBP induced a high rate (>60%) of cryptorchidism (mainly unilateral), hypospadias, infertility, and testis abnormalities similar to those observed in human testicular dysgenesis syndrome. They hypothesised that abnormal development of Sertoli cells was the explanation for the abnormal changes in DBP-exposed rats. However, these effects were not observed in the mouse model [36]. Mice appear to be resistant to in utero phthalate-induced foetal testis endocrine disruption. The effects of in utero phthalate exposure therefore appear to be species-dependent. Habert et al. [37] studied in vitro responses to six EDs in rats, mice, and humans, and showed differences in susceptibility to one third of the compounds tested between human and rodent foetal testes. They emphasized the need to develop specific tools to study reproductive toxicity in humans. The identification of common molecular targets in both rat and human models is necessary before selecting the toxicological endpoint used in rats to accurately assess the safety threshold of EDs in humans.

Contradictory evidence has been reported for the effects of phthalates in humans. On the one hand, according to Lottrup et al. [38], two recent studies indicated that human testicular development might be susceptible to phthalates. One study analysed phthalate monoesters in breast milk and reproductive hormone levels in infants. Five phthalates were correlated with hormone levels in healthy boys, which were indicative of lower androgen activity and reduced Leydig cell function. Another study found a reduction of the anogenital index (AGI) in infant boys with increasing levels of phthalates in maternal urine samples during late pregnancy. Boys with low AGIs showed a high prevalence of cryptorchidism and small genital size. Taken together, these studies suggest an antivirilising effect of phthalates in infants. Phthalates appear to exert an adverse effect on the function of Leydig cells in the testis, decreasing androgen production. Antenatal exposure to phthalates during a critical window corresponding to the hormone-dependent period of development of the male genitalia could promote genital anomalies. On the other hand, some human in vitro and in vivo studies found no effect [39,40] or no significant effects [41] of exposure of the human foetal testis to phthalates.

Moreover, exposure to mixtures of phthalates and other endocrine-disrupting compounds could additively contribute to these adverse effects. In particular, phthalates and bisphenol A are suspected to interfere with the endocrine system. Christen et al. [42] analysed the in vitro antiandrogenic activity of mixtures of phthalates with and without bisphenol A. A mixture of five phthalates, representing a human urine composition and reflecting exposure to corresponding parent compounds, showed no antiandrogenic activity. This study demonstrated that concentration addition (CA) is an appropriate concept to account for mixture effects of antiandrogenic phthalates and bisphenol A. The interaction indicates a departure from additive antagonism at low concentrations, probably due to interactions with the androgen receptor and/or cofactors. This study emphasised that a risk assessment of phthalates should take mixture effects into account.

Although the results are mainly derived from in vitro and animal studies, there is accumulating evidence of the impact of phthalates on genital development during prenatal exposure. Further studies are necessary to shed light on the possible mechanisms of action of developmental effects caused by phthalates in humans.

Some results of univariate analysis in this study also suggested a possible role of EDCs in the home environment, as more women in the Hypospadias Group owned a garden or used veterinary insecticides. Parents could be exposed to EDCs via inhalation or dermal contact with plant or pet treatments. More women in the Hypospadias Group declared living close to a field. Despite experimental evidence suggesting that pesticides may be associated with hypospadias, there is no evidence to suggest that living close to sites of pesticide application constitutes a real risk factor [43,44,45]. Geolocalisation data could be used to refine these results.

### 4.4. Occupational EDCs Exposure

We used a validated JEM for EDCs [8] created to address the specific issue of the impact of parental occupational exposure to potential EDCs on the risk of hypospadias in their offspring. As emphasised by Nassar et al. [46], coding parental occupations can be a source of bias. Although the matrix is very specific, job titles do not always strictly comply with the list of 348 job descriptions. This potential coding bias was minimised in our study by using double, blinded coding.

Multivariate analysis demonstrated that the risk of hypospadias was strongly associated with maternal occupational exposure to EDCs. Nassar et al. [46] found an association with potential maternal exposure to heavy metals and a possible association with phthalates. According to Kalfa et al. [2], occupational EDCs exposure was more frequently observed among mothers of boys with hypospadias than among mothers of control boys. Sample sizes were too small to demonstrate any statistically significant impact of specific pollutants.

Some jobs seem to be at greater risk of exposure to EDCs, such as cleaners, hairdressers, painters, beauticians, and laboratory workers [2,28]. However, this trend was not observed in our study due to the wide range of maternal occupations. Vrijheid et al. [28] found little evidence for a relationship between hypospadias and maternal occupation, but an increased risk of hypospadias in the offspring of hairdressers and occupations exposed to phthalates. Kalfa et al. [2] highlighted the potential cumulative effect of various exposures. Our results also support the role of a mixed exposure in the incidence of hypospadias.

This study demonstrated two independent risk factors for hypospadias linked with occupational and household exposure. Current evidence supports the hypothesis of a multifactorial aetiology of male genital malformations [47]. It is highly probable that mixtures of EDCs have additive effects, which has been described as a “cocktail” effect. In animal studies, Rider et al. [48] compared the cumulative effects of in utero administration of mixtures of reproductive toxicants that disrupt common target tissues via various mechanisms of toxicity. These compounds, regardless of their specific mechanism of action, display cumulative dose-additive effects when present in combination. Kortenkamp et al. [49] reviewed the literature on low-dose mixtures and found good evidence demonstrating significant mixture effects with combinations of chemicals well below their individual “no observable adverse effect” levels (NOAELs), with mixtures composed of agents with both similar and dissimilar mechanisms of action. Similarly, Vandenberg et al. [50] reported that EDCs can exert effects at low doses that are not predicted by the effects observed at higher doses due to the existence of nonmonotonic dose–response curves of EDCs. Lastly, Carruthers and Foster [51] identified a critical window of male reproductive tract development in rats following gestational exposure to phthalates. The timing of exposure also appears to be a critical component in the adverse outcomes of EDCs exposure.

## 5. Conclusions

Our findings provide evidence of an association between maternal exposure to EDCs during early pregnancy and an increased risk of hypospadias in the offspring. The effects of occupational exposure to EDCs remain controversial. For the first time, the use of hair cosmetics at home has been identified as a risk factor for hypospadias. A larger study is needed to more clearly determine the risks associated with the use of cosmetics. Considerable efforts have been made over recent years to test and identify the effects of EDCs, but their effects on the offspring remain difficult to determine. EDCs including phthalates are strongly suspected to participate in the pathophysiology of hypospadias and TDS; our results support this hypothesis. Until the effects of EDCs have been more clearly determined, the precautionary principle [52] should apply to pregnant women; in particular, they should be advised to limit their use of hair cosmetics.

## Figures and Tables

**Table 1:** Severity of hypospadias and description of congenital defects in our population.

	Cases (*n* = 57)	Controls (*n* = 162)
Severity of hypospadias	48 (84%) distal (26 glandular, 14 coronal, 8 distal shaft) 6 (11%) middle 3 (5%) proximal (1 penoscrotal, 2 perineal)	-
Twins	5	4
Congenital defects	10.5% Undescended testis (OR: 5.8, 95% CI: 1.4–8.2) 0 Micropenis 2 VACTEL syndrome + undescended testis 1 Anorectal malformation and oesophageal atresia 1 Vertebral and cardiac malformations	2% 0
		0

OR: odds ratio; 95% CI: confidence interval; VACTEL: vertebral abnormalities, anal atresia, cardiac abnormalities, tracheoesophageal fistula, limb defects syndrome.

**Table 2:** Univariate analysis of the association between neonatal risk factors and the incidence of hypospadias.

		Cases (*n* = 57) Mean ± SD (*n)*	Controls (*n* = 162) Mean ± SD (*n)*	*p*-Value OR (95% CI)
Term (WA)		38.3 ± 1.82 (56)	39.3 ± 1.5 (160)	0.26
Weight (g)		3100 ± 561 (57)	3308 ± 429 (162)	<0.01 **
Length (cm)		48.2 ± 2.8 (57)	49.6 ± 2.2 (148)	<0.001 ***
HC (cm)		34.3 ± 1.8 (57)	34 ± 1.4 (161)	0.21
Low birth weight ^§^	Yes No	9 56	4 158	<0.001 *** 7.4 (2.2–25.1)
Low birth length ^§^	Yes No	9 56	7 143	<0.01 ** 3.8 (1.4–10.8)
First born	Yes No	27 30	51 105	0.03 * 2.0 (1.1–3.7)

* *p* < 0.05; ** *p* < 0.01; *** *p* < 0.001. ^§^ Gestational age adjusted for mother’s age and baby’s gender below the 5th percentile. SD: standard deviation; WA: weeks of amenorrhea

**Table 3:** Univariate analysis of the association between parental risk factors and the incidence of hypospadias.

		Cases (*n* = 57) Mean ± SD (*n)*	Controls (*n* = 162) Mean ± SD (*n)*	*p*-Value OR (95% CI)
**Maternal Factors**				
**Biometric parameters**				
Age (year)		29.7 ± 5.4 (56)	28.7 ± 5.5 (161)	0.24
Age > 40 years (*n*)	Yes No	4 52	7 155	0.4 1.7 (0.5–6.0)
Weight (kg)		64.5 ± 15.3 (51)	67.9 ± 17.3 (147)	0.22
BMI (kg/cm^2^)		23.9 ± 5.5 (54)	24.7 ± 5.7 (150)	0.43
**Medication**				
Medication (*n*)	Yes No	13 38	21 131	0.05 * 2.1 (1.0–4.6)
Folate (*n*)	Yes No	10 36	47 86	0.07 0.5 (0.2–1.1)
Contraception (*n*)	Yes No	37 10	114 27	0.75 0.9 (0.4–2.0)
Pill (*n*)	Yes No	31 14	101 59	0.21 1.2 (0.6–2.6)
**Endocrine medical history**				
History of miscarriages (*n*)	Yes No	15 36	44 105	1.0 (0.5–2.0)
Age of menarche (*n*)		12.9 *±* 1.7 (144)	12.9 ± 1.5 (37)	1.0
Smoking during pregnancy (*n*)	Yes No	16 35	50 101	0.8 0.9 (0.5–17.8)
**Social level**				
Maternal education	Low High	30 16	70 66	0.12 0.6 (0.3–1.2)
**Family Medical History**				
Undescended testis (*n*)	Yes No	2 45	14 99	0.12 NA
Fertility disorders (*n*)	Yes No	1 47	0 111	0.30 NA
Hypospadias (*n*)	Yes No	5 44	3 110	0.04 * NA
**Paternal Factors**				
Age (year)		32.5 ± 6.5 (51)	31.4 ± 6.8 (141)	0.16
Age > 40 years (*n*)	Yes No	9 41	15 126	0.18 1.8 (0.7–4.5)
Weight (kg)		77.6 ± 12.4 (46)	83.4 ± 13.5 (158)	0.03 *
BMI (kg/cm^2^)		24.6 ± 3.4 (35)	26.3 ± 4.4 (159)	0.04 *
Median BMI (25.3 kg/cm^2^)	<25.3 >25.3	21 24	36 43	0.15 1.79 (0.8–4.0)

* *p* < 0.05; NA: not applicable; BMI: body mass index.

**Table 4:** Univariate analysis of the association between pollutant exposures during the first trimester of pregnancy and the incidence of hypospadias.

		Cases (*n* = 57)	Controls (*n* = 162)	*p*-Value, OR (95% CI)
**Cosmetics**				
Hair cosmetics	Yes No	16 35	51 83	0.07 1.8 (1.0–3.6)
-Hairspray	Yes No	16 31	37 95	0.4 1.3 (0.6–2.7)
-Colouring shampoo	Yes No	13 34	25 105	0.2 1.3 (0.7–3.5)
**Chemicals**				
Chemicals	Yes No	41 6	120 11	0.38 0.6 (0.2–1.8)
-Paint/solvents/gasoline	Yes No	5 42	23 108	0.3 0.6 (0.2–1.6)
-Ink	Yes No	3 44	6 124	0.6 NA
-Glue	Yes No	5 42	13 118	0.90 1.1 (0.4–3.2)
-Household products	Yes No	41 5	120 11	0.6 0.8 (0.2–2.3)
Human antiparasitic	Yes No	13 38	57 93	0.10 0.56 (0.3–1.1)
**Environmental Factors**				
Living <1 km from a field	Yes No	33 14	76 61	0.07 1.9 (0.9–3.8)
Living <1 km from a factory	Yes No	12 31	43 78	0.3 0.7 (0.3–1.5)
Garden	Yes No	40 8	99 46	0.04 * 2.3 (1.0–5.3)
Pets	Yes No	38 13	85 65	0.02 * 2.2 (1.1–4.5)
Veterinary insecticides	Yes No	37 14	84 65	0.04 * 2.0 (1.02–4.1)
**Occupational Factors**				
Working during pregnancy	Yes No	32 19	86 65	0.6 1.3 (0.6–2.3)
Occupational exposure to EDCs (JEM)	Yes No	11 44	12 150	0.007 ** 3.1 (1.3–7.6)

* *p* < 0.05; ** *p* < 0.01.

**Table 5:** Multivariate model of the association between maternal exposure and the incidence of hypospadias.

Risk Factors	OR	95% CI	*p*-Value
Medication (yes vs. no)	2.34	0.30–18.05	0.41
Human insecticides	2.69	0.49–14.63	0.25
Veterinary insecticides	1.56	0.25–9.64	0.63
Household use of hair cosmetics	6.11	1.07–34.86	0.04
Living <1 km from a field (yes vs. no)	3.51	0.38–32.31	0.27
Occupational exposure to EDCs (JEM)	9.64	1.41–66.09	0.02

OR adjusted for neonatal birth weight, parity, paternal weight, maternal folate intake. *p* < 0.05 is underlined.
