# Erratum: Haraux, E., et al. Maternal Exposure to Domestic Hair Cosmetics and Occupational Endocrine Disruptors Is Associated with a Higher Risk of Hypospadias in the Offspring. *Int. J. Environ. Res. Public Health* 2017, *14*, 27

**DOI:** 10.3390/ijerph14090989

**Published:** 2017-08-31

**Authors:** Elodie Haraux, Karine Braun, Philippe Buisson, Erwan Stéphan-Blanchard, Camille Devauchelle, Jannick Ricard, Bernard Boudailliez, Pierre Tourneux, Richard Gouron, Karen Chardon

**Affiliations:** 1 Department of Paediatric Surgery, Amiens University Hospital, 80054 Amiens, France; buisson.philippe@chu-amiens.fr (P.B.); ricard.jannick@chu-amiens.fr (J.R.); gouron.richard@chu-amiens.fr (R.G.); 2 Department of Paediatrics, Amiens University Hospital, 80054 Amiens, France; braun.karine@chu-amiens.fr (K.B.); boudailliez.bernard@chu-amiens.fr (B.B.); 3 PériTox-INERIS Laboratory, Jules Verne University of Picardy, 80054 Amiens, France; erwan.stephan@u-picardie.fr (E.S.-B.); karen.chardon@u-picardie.fr (K.C.); 4 Department of Paediatrics, Creil Hospital, 60100 Creil, France; camille.devauchelle@ch-creil.fr; 5 Department of Paediatric Intensive Care Unit, Amiens University Hospital, 80054 Amiens, France; tourneux.pierre@chu-amiens.fr

Due to an error during production, some data presented in Table 4 in the Experimental section of the published paper [1] were incorrect. A corrected version of this table is provided below. Importantly, these changes do not modify the significance of the results and the related conclusions. The authors would like to apologize for any inconvenience caused to the readers by this error. The article will be updated and the original will remain on the article webpage.

## Figures and Tables

**Table 4:** Univariate analysis of the association between pollutant exposures during the first trimester of pregnancy and the incidence of hypospadias.

		Cases (*n* = 57)	Controls (*n* = 162)	*p*-Value, OR (95%CI)
**COSMETICS**				
**Hair cosmetics (*n*)**	Yes	25	51	0.07
	No	22	81	1.9 (0.9–3.5)
**- Hairspray (*n*)**	Yes	16	37	0.4
	No	31	95	1.3 (0.6–2.7)
**- Colouring shampoo (*n*)**	Yes	13	25	0.2
	No	34	105	1.6 (0.7–3.5)
**CHEMICALS**				
**Chemicals (*n*)**	Yes	41	120	0.38
	No	6	11	0.6 (0.2–1.8)
**- Paint/solvents/gasoline (*n*)**	Yes	5	23	0.3
	No	42	108	0.6 (0.2–1.6)
**- Ink (*n*)**	Yes	3	6	0.6
	No	44	124	NA
**- Glue (*n*)**	Yes	5	13	0.90
	No	42	118	1.1(0.4–3.2)
**- Household products (*n*)**	Yes	41	120	0.6
	No	5	11	0.8 (0.2–2.3)
**Human antiparasitic (*n*)**	Yes	13	57	0.10
	No	38	93	0.56 (0.3–1.1)
**ENVIRONMENTAL FACTORS**				
**Living < 1 km from a field**	Yes	33	76	0.07
	No	14	61	1.9 [0.9–3.8]
**Living < 1 km from a factory**	Yes	12	43	0.3
	No	31	78	0.7 (0.3–1.5)
**Garden (*n*)**	Yes	40	99	**0.04** *
	No	8	46	2.3 (1.0-5.3)
**Pets (*n*)**	Yes	38	85	**0.02** *
	No	13	65	2.2 (1.1–4.5)
**Veterinary insecticides (*n*)**	Yes	37	84	**0.04** *
	No	14	65	2.0 (1.02–4.1)
**OCCUPATIONAL FACTORS**				
**Working during pregnancy (*n*)**	Yes	32	86	0.6
	No	23	76	1.2 (0.7–2.3)
**Occupational exposure to EDCs (JEM) (*n*)**	Yes	11	12	**0.007** **
	No	44	150	3.6 (1.4–9.3)

* *p* < 0.05; ** *p* < 0.01.
